# Alpha, Beta, gamma human PapillomaViruses (HPV) detection with a different sets of primers in oropharyngeal swabs, anal and cervical samples

**DOI:** 10.1186/s12985-019-1132-x

**Published:** 2019-03-04

**Authors:** Catia Sias, Leonidas Salichos, Daniele Lapa, Franca Del Nonno, Andrea Baiocchini, Maria Rosaria Capobianchi, Anna Rosa Garbuglia

**Affiliations:** 1 Laboratory of Virology, “Lazzaro Spallanzani” National Institute for Infectious Diseases, IRCCS, Via Portuense, 292, 00149 Rome, Italy; 2 Laboratory of Pathology, “Lazzaro Spallanzani” National Institute for Infectious Diseases, IRCCS, Rome, Italy; 3 Program in Computational Biology and Bioinformatics, Yale University, New Haven, CT 06520 USA

**Keywords:** Human papillomaviruses, Betapapillomavirus, Gammapapillomavirus, HPV diagnosis, HPV detection methods, Oropharyngeal HPV infection

## Abstract

**Background:**

Recent studies have shown a 13-fold increase of oropharyngeal cancer in the presence of HPV, while α-HPV detection seems to be rare in oral cavity in comparison to anal or cervical district, many novel β and γ types have been isolated in this anatomical site suggesting a wide tropism range. Currently, there are no guidelines recommending HPV oral cavity screening as a mandatory test, and it remains unknown which HPV types should be included in HPV screening programs. Our goal was to assess HPV prevalence in oropharyngeal, anal, and cervical swabs using different sets of primers,which are able to amplify α, β, γ HPV types.

**Methods:**

We analysed the presence of HPV DNA in oropharyngeal (*n* = 124), anal (*n* = 186), cervical specimens (*n* = 43) from HIV positive and negative patients using FAP59/64 and MY09/11 primers. All untyped strains were genetically characterized through PCR amplification and direct sequencing of partial L1 region, and the resulting sequences were classified through phylogenetic analysis.

**Results:**

HPV prevalence was 20.9% in 124 oropharyngeal swab samples, including infections with multiple HPV types (5.6%). HPV prevalence in this anatomical site was significantly associated with serostatus: 63.3%in HIV positive and 36.3% in HIV negative patients (*p* < 0.05).

Unclassified types were detected in 6 specimens. In our analysis, we did not observe any difference in HPV (α, β, γ) prevalence between men and women. Overall, β species were the most frequently detected 69.7%. When using anal swabs, for HIV positive patients, β genus prevalence was 1% and γ genus was 3.7% including 6 unclassified types. In cervical samples from 43 HIV positive women (18 HPV negative and 25 positive by MY09/11 PCR), only one sample was positivite for β_1_ species (2.4%) using FAP primers. Six of the untyped strains clustered with sequences from species 7, 9, 10, 8,12 of γ genus. Four sequences remained unclassified. Finally, β and γ HPV prevalence was significantly lower than their respective HPV prevalence as identified by the Luminex system in all anatomical sites that were analyzed in previous studies.

**Conclusion:**

This study provides new information about viral isolates present in oropharyngeal site and it will contribute to improve the monitoring of HPV infection.

**Electronic supplementary material:**

The online version of this article (10.1186/s12985-019-1132-x) contains supplementary material, which is available to authorized users.

## Introduction

Human papillomavirus (HPV) persistent infections are considered the primary cause of ano-genital cancer, where greater than 99% of cervical cancer and more than 60% of anal cancer contain HPV DNA [1, 2]. Throughout the past years, several studies have suggested the link between HPV infection and other epithelial cancers, including cutaneous and oropharyngeal cancers. Oropharyngeal cancers are often referred to as “head and neck cancer” (HNC), and mainly include squamous-cell carcinoma which occurs in the oral cavity, base of tongue, tonsils, adenoids pharynx and larynx. Recent studies on HPV infection in oral exfoliated cells have shown that there is a 13-fold higher risk for oropharyngeal cancer in the presence of virus [3–5]. The highest prevalence (up to 50%) for these HPV associated cancers has been found in the tonsillar cancers sub-set [6–8]. Since HPV16 presented a global prevalence in oropharyngeal squamous cell carcinoma (OPSCC) and oral squamous cell carcinoma (OSCC) of 40.6 and 14.9% respectively [9], IARC categorized this genotype as a risk factor with for the pathogenesis of both cancers [10]. Additionally, other HPV types have also been linked with these cancers, including HPV18, 31, and 33 [11] including β and ɣ types [4]. Furthermore, while α-HPV detection seems to be rare in oral cavity in comparison to anal or cervical districts [12], many novel β and γ types have been isolated in oral cavity [13] suggesting a wide tropism range of these genera [14]. At the same time, different studies on HPV prevalence provided contradicting result for β and ɣ types not only in oropharyngeal site, but also in cervical and anal anatomical sites [14–19].

To date, there are no guidelines recommending HPV oral cavity screening as a mandatory test, and no HPV DNA test has been approved for HPV detection in oral cavity. In addition, it remains unknown which HPV types should be included in oral cavity screening. Agalliou [4] highlighted the link between β, γ HPV types and oral cancers, assessing the presence of HPV types which are not generally detected with commercial assays in cervical cancer screening. Even though different methods have been used to detect novel HPV types, the association between new β and γ HPV types and oral cavity cancer has not yet been established.

In this study, we analyzed the presence of HPV DNA in oral, anal, and cervical specimens collected from HIV positive and HIV negative individuals, living in the same geographic area (regione Lazio) by using MY09/11 [20, 21] FAP59/64 primers [22]. These primers, both targeting highly conserved region within L1 ORF, are considered broad range PV primers and allow the detection of the great majority of already known and officially recognized HPV types. MY09/11 had been used in α HPV detection in cervical sites, where they showed good sensitivity in alpha HPV types amplification [21], while FAP primers officially recognized HPV from α-PV, β-PV, and ɣ-PV genera, and might detect potentially new types [22–25]. This property is particularly relevant, since ɣ and β papillomaviruses have been already identified in several anatomic sites [14, 26]. Granted that that HPV prevalence is significantly higher among HIV infected people and at multiple anatomic sites [26–29] we included both HIV positive and HIV negative people in our study in order to also verify whether β and ɣ HPV positivity is influenced by host immune status.

## Materials and methods

### Study population and sample collection

This is a retrospective study carried out at Laboratory of Virology INMI L Spallanzani on residual oropharyngeal samples collected for respiratory virus detection, anal and cervical swabs collected for HPV testing in diagnostic routine. The Hospital ethics committee approved the protocol. Date of birth, date of swabs sampling, HIV serostatus were recorded from Institutional database. In HIV positive patients, the most recent CD4 T cell count (± 1 month from the date of sample collection) and HIV RNA viral load (± 1 month from the date of sample) with a detection limit of 40cp/ml (Abbott Molecular Inc., Des Plaines, Il, USA) were used to correlate clinical features and HPV positivity.

Oropharyngeal swabs -Oropharyngeal samples were collected as following: the nylon-flocked tip was rotated 3–4-times against right and left buccal mucosa, palatine, tonsils, upper and lower pharynx area. The swab was then plunged and stirred in 1.5 mL DMEM medium with streptomycin and ampicillin. Specimens were refrigerated within 3–5 h after collection until processing.

Anal swabs -We retrospectively analysed 186 anal swabs previously tested by MY09/11 primers and typed by CLART HPV2 Clinical array or Sanger sequencing (see HPV detection and typing section, below). One hundred samples belonged to HIV positive women (50 HPV positive by MY09/11 PCR and 50 negative by MY09/11 PCR), 86 anal swabs were collected from men who have sex with men (MSM). All MSM specimen resulted positive in MY09/11 PCR.

Cervical swabs *-* A total of 43 cervical swabs (18 HPV negative and 25 HPV positive, assigned by MY09/11 PCR) were considered in this study. All samples belonged to HIV positive women.

General characteristics of HIV infected patients are presented in Additional file 1: Table S1.

### DNA isolation

Oropharyngeal swabs were removed from the medium which we divided in two parts: half part was used for detecting respiratory viruses panel (Influenza-A, B, RSV, rhinovirus, coronaviruses, metapneumovirus, adenovirus) and the other half was employed in testing HPV DNA, having been stored at − 80 °C until use. Before nucleic acid extraction, all specimens were pre-treated. Briefly, 600 μL of clinical material was digested with 20 μL proteinase K solution (QIAGEN, Hilden Germany) and lysed with AL lysis buffer (QIAGEN, Hilden, Germany) at 56 °C for 10 min. Nucleic acid extraction was done with a magnetic bead-based automated platform (QIASYMPHONY, Hilden, Germany) in accordance with the manufacturer’s instructions. Nucleic acids were eluted in 60 μL of AVE elution buffer (QIAGEN, Hilden, Germany). Nucleic acid from anal and cervical swabs were extracted as described elsewhere [30, 31].

Samples that were β-globin negative were excluded from the study [32, 33].

### Human papillomavirus detection and genotyping

Ten μL of eluted nucleic acids were employed for evaluating the presence of HPV types by using MY09(5’CGTCCMARRGGAWACTGATC3’) and MY11(5’GCMCAGGGWCATAAYAATGG3’) [20, 21] PCR and FastStart DNA polymerase (Roche Diagnostics GmbH, Mannheim, Germany). The PCR assay conditions were: 95 °C for 5 min, then 39 cycles (denaturation 95 °C/30 s, annealing 55 °C/45 s, and extension 72 °C/1 min). One last step for extension was employed at 72 °C for 10 min. Fifteen μL of the PCR products were mixed with 6 loading solution in 1.8% agarose gel electrophoresis stained by ethidium bromide and run for 30 min at 130 V. HPV genotyping of positive samples was conducted using Genomica Clinical HPV array (Genomica, Madrid, Spain). CLART HPV2 clinical array HPV is able to detect: 6, 11, 16, 18, 26, 31, 33, 35, 39, 40, 42, 43, 44, 45, 51, 52, 53, 54, 56, 58, 59, 61, 62, 66, 68a, and b, 70, 71, 72, 73, 81, 82, 83, 84, 85, and 89.

All adequate samples were retested using FAP 59 (5’TAACWGTIGGICAYCCWTATT3*’)* and FAP 64 (5*’*CCWATATCWVHCATITCICCATC*3’)* primers [22] able to detect α, β and γ HPV types. FastStart DNA polymerase were used in this assay condition: 5 min at 94 °C, 40 cycles (denaturation 94 °C/30 s, annealing 52 °C/45 s, and extension 72 °C 1 min) followed by a final extension step at 72 °C for 7 min. PCR products were also run on agarose gel. Positive samples with FAP set of primers and those positive with MY09/11 PCR protocol, but negative in Genomica typing assay were purified and sequenced on the automated ABI Prism 3100 instrument, by using a BigDye Terminator cycle sequencing kit (Applied Biosystems, Warrington, UK).

### Phylogenetic analysis

HPV sequences were aligned using mafft [34] using the L-INS-I method. After visual inspection using Seaview [35] we removed sample sequence Q1359 and Q1017 were excluded from the alignment because they represented mixed infection and the Sanger sequence interpretation was not optimal. Reference sequences HM999999_HPV147 and GU129016_HPV148 were also excluded from the alignment. Then, we further implemented gblocks [36] to remove ambiguous positions using the least stringent options. To infer a maximum likelihood tree, we implemented RAxML [37] using a GTRCAT model, with 100 bootstrap replicates. Tree visualization was achieved using Figtree [38]. Finally, pairwise similarity matrices were constructed using BLAST.

## Results

### HPV detection in oropharyngeal swabs, anal and cervical samples

Oropharyngeal swabs-A total of 124 oropharyngeal swabs with β-globin positive signal were considered in this study. HPV prevalence was 20.9% (26/124), including infections with multiple HPV types (7/124, 5.6%) (Table 1). MY09/11 PCR gave positive results in 18 samples. However, a BLAST search against GenBank indicated that 5 amplified fragments were identical to human sequences (5/124, 4%), thus true HPV positive rate was estimated at 10.5%.

**Table 1:** HPV types detected with 2 different primers set in oropharyngeal swabs from HIV positive and HIV negative patients

	Sample code (sex)	MY09/11PCR	FAP59/64 PCR
HIV positive patients	Q200 M		HPV19(β_1_)
	Q227 F	HPV145(β_2_)	HPV19(β_1_)
	Q255 F	HPV110(β_2_)	
	Q266 F	HPV145(β_2_)	HPV5(β_1_)
	Q447 F		HPV5(β_1_)
	Q532 F		HPV132(γ_12_)
	Q581 M		HPV5(β_1_)
	Q637 F		HPV5(β_1_)
	Q656 F		§
	Q686 M		HPV19(β_1_)
	Q947 F	HPV70(α_7_)	
	Q996 F	HPV16(α_9_)	
	Q1017 F	HPV120(β_2_)	§
	Q1234 F	HPV17(β_2_)	§
	Q1644 F		§
	Q1766 M	HPV17(β_2_)	§
HIV negative patients	Q127 M	HPV145(β_2_)	§
	Q425 F		HPV8(β_1_)
	Q718 F		HPV19(β_1_)
	Q739 M	HPV120(β_2_)	
	Q760 M	HPV145(β_2_)	HPV20(β_1_)
	Q1133 M	HPV17(β_2_)	
	Q1643 M		HPV19(β_1_)
	Q1657 M		HPV20(β_1_)
	Q1833 M		HPV5(β_1_)
	Q2050 M	HPV13(α_10_)	

§ untyped HPV strains; *F*, female, *M*, male

Out of 13, three samples were identified as α and ten as β types. Alpha-types were detected in two HIV positive patients (2/55, 3.6%), and in 1 HIV negative patient. HPV α-types, 16 and 70, were detected in HIV positive subjects (2/55, 3.6%) after being typed by CLART HPV2 clinical array, while HPV13 was amplified in HIV negative subject (1/69, 1.4%); it was typed by Sanger sequencing. All β HPV types belonged to β_2_ species (Table 1), and HPV145 was the most frequent type (4/10), both in HIV positive and HIV negative patients; 20 samples were identified as positive by FAP PCR. No specific amplicons were observed. Seven FAP-detected HPV types were found in samples that also gave positive results with MY09/MY11 PCR (Table 1). Types β_1_ were the most represented (*n* = 13), while one sample was identified as γ type (HPV132, γ_12_). Additionally, one mixed infection (Q1017) and 5 untyped HPV strains were also detected. Among β_1_ species, the most frequent types were HPV5 (*n* = 5, 38.6%) and HPV19 (n = 5, 38.6%). Three multiple infections harboured β_1_ and β_2_ types, whereas β_2_ types and untyped strains were observed in 4 other multiple infections. No multiple infection harboured α with β or γ species (Table 1). Overall, β species were most prevalent (*n* = 23/33, 69.7%). HPV distribution differed significantly when the number of HPV β, γ-types were considered in HIV positive and HIV negative subjects: 21 (21/33, 63.6%) vs 12 (12/33, 36.3%) respectively (Chi square test, *p* < 0.005). HIV positive people with HPV in oropharyngeal swabs showed a mean CD4 T cell count of 532.3 ± 275.6. Among HIV positive patients with detectable HPV, 6/16 had no detectable HIV RNA in plasma and the other patients (*n* = 10) showed a mean RNA copies/mL169.0 ± 130.0.

HPV prevalence rates were compared for statistical significance, using a Chi squared test, in men and women both HIV positive and HIV negative. However, no difference was observed (Chi square test, *p* > 0.05).

Anal swabs- Eighty-six anal swabs from MSM resulted positive by MY09/11 PCR, and 100 anal swabs from women (50 HPV positive by MY09/11 PCR and 50 negative by MY09/11 PCR) were retested with FAP primers.

Among MSM anal swabs, 15 samples showed single HPV infection by CLART array testing, and 71 patients harboured multiple HPV infection with at least one high risk (HR) type. In 61.2% of samples we observed a clinically evident anal pathology, including 7 subjects with anal intraepithelial neoplasia (AIN) II, 46 patients with AIN I. Among HPV positive women, 18 harboured a single infection (AIN II, *n* = 0; AIN I, *n* = 5); atypical squamous cells of undetermined significance (ASC-US), n = 0; normal, *n* = 13) and 31 samples showed HPV multiple infection, all with at least 1HR type (62%) (AIN 2,n = 0; AIN I, *n* = 9; normal cytology, *n* = 22). HPV type was undetermined in one sample. Among single infection with AIN I, one specimen was infected with a low risk (LR)Type (HPV81). Nineteen anal swab specimens were identified as HPV positive by FAP primers detection (female anal swabs, *n* = 3; male anal swabs, *n* = 16) (Table 2). Overall α-type strains were detected in 9 samples with FAP primers which were not previously detected by MY09/11 primers. All but one α-type strains were also detected with α mucosal HPV multiple infection typed with CLART HPV2 clinical array which included at least one HR type. HPV β_2_ types (HPV9 and HPV37) were found in two specimens co-infected with α HPV types; 2 specimens harboured γ_10_ HPV types (HPV121 and HPV180), both associated with type α HPV multiple infections. All FAP untyped strains (*n* = 6) were found in anal specimens infected by α types with at least one high HR type. Considering cytological aspects, 12/19 samples, which resulted positive with FAP primer PCR, had AIN (I, II) lesions, while 7/19 samples showed normal cytology (Table 2).

**Table 2:** Different HPV genera detected in male and female anal swabs by FAP primers

Patient code	Sex	PCR by FAP primer	HPV genotypes typed by Genomica	Cytological lesion	CD4T cell count*	HIVRNA Viral load ^
	**α-1**					
Q74	M	HPV 32	6; 16; 42; 45; 61; 52; 53; 59	AIN I	482	Detected < 40 cp/ml
Q497	F	HPV 32	6; 31; 42; 53	AIN I	258	95
	**α-3**					
Q802	F	HPV114	16	Negative	986	No detected
	**α-6**					
Q1420	M	HPV30	16; 70; 71; 83	AIN II	372	Not detected
Q1664	M	HPV30	44; 51; 59; 81	Negative	897	118
Q1958	M	HPV30	33; 56; 58; 72	Negative	226	Detected < 40 cp/ml
	**α-8**					
Q1224	M	HPV43	11; 40; 68	AIN I	421	Not detected
	**α-14**					
Q2167	M	HPV90	33; 61; 72	AIN I	797	Detected < 40 cp/ml
Q1211	F	HPV90	35; 51; 84	AIN I	372	Detected < 40 cp/ml
	**β-2**					
Q324	M	HPV9	6; 53; 66	AIN I	453	Not detected
Q1420	M	HPV37	31; 70	Negative	919	Not detected
	**γ-10**					
Q1652	M	HPV121	6; 58	AIN I	383	799
Q1661	M	HPV180	31; 44; 56; 70	Negative	N.D.	N.D.
	***Unclassified***					
Q654	M	SE17	11; 31; 44; 51; 58; 62; 66	AIN I	612	362
Q760	M	FA79	35; 44; 66; 81	AIN I	601	185
Q763	M	FAIMVS9	33; 58; 70; 81	AIN I	392	Not detected
Q1539	M	FA138	53; 66;84; 85	Negative	886	111
Q2164	M	FA39	16; 33	Negative	648	Not detected
Q1354	M	MM7	31; 45	AIN I	630	Not detected

*HPV* human papillomavirus; *cell/μl; ^ copies/ml; *N.D.* not done

Among anal swabs from females (*n* = 100), only 3 samples (3.0%), previously tested MY09/11 HPV positive, resulted HPV positive with FAP primer (HPV32, α_1_ species; HPV114, α_3_ species; HPV90, α_14_ species). No untyped HPV strains were observed among female samples.

Overall, β genus prevalence was 1% and γ genus was 3.7% including untyped isolates.

Mean of CD4 T cell count was 690.7 ± 343.9 cells/μL. Overall 170/186 patients (91.4%) were under antiretroviral therapy (ART). All patients without ART had a CD4 T cell count > 400/ μL. Current ART use was not associated with risk of β and ɣ infection (Chi square test, *p* > 0.05).

Cervical swabs-Twelve specimens showed a single HPV infection, and 13 had multiple infections by CLART HPV2 array; 17/25 samples harboured at least 1 HR genotype.

Cytology findings were available for 20 specimens already resulted positive by CLART HPV2 clinical array: 10 had normal cytology, 6 were ASC-US or low grade squamous intraepithelial lesion (LSIL), 3 harboured HPV single infections, 3 showed HPV multiple infections, and 4 were high grade squamous intraepithelial lesion (HSIL). All MY09/11 HPV negative women (*n* = 18) showed normal cytological findings. Eleven/43 cervical samples resulted FAP positive (25.6%). However, only 6 HPV types were not previously detected by MY09/11 primers. Five samples harboured α types (HPV90, *n* = 3 α_14_; HPV32, n = 1, α_1_; HPV68 α_7_, n = 1), and only one specimen gave positivity for β_1_ (HPV14) (2.4%). The mean CD4 T cell count was 539 ± 230 cells/μL. Ninety-six percent of women were receiving ART per WHO guideline of starting ART at CD4T cell count < 350 cell/mm^3^. The woman harboring β HPV strain was under ART. She had a CD4 T cell count of 540 cells/μL and HIV RNA viral load was not detected.

Overall cervical and anal β and γ type positivity was not related to immune status as measured by CD4 T cell count (see Table 2) and ART treatment. Furthermore, the immune-re constitution could potentially prevent the persistence of β and ɣ types in anal and cervical sites or alternatively, β and ɣ types might show to have a less tropism to these anatomical sites.

### Phylogenetic analysis

**Fig. 1:**
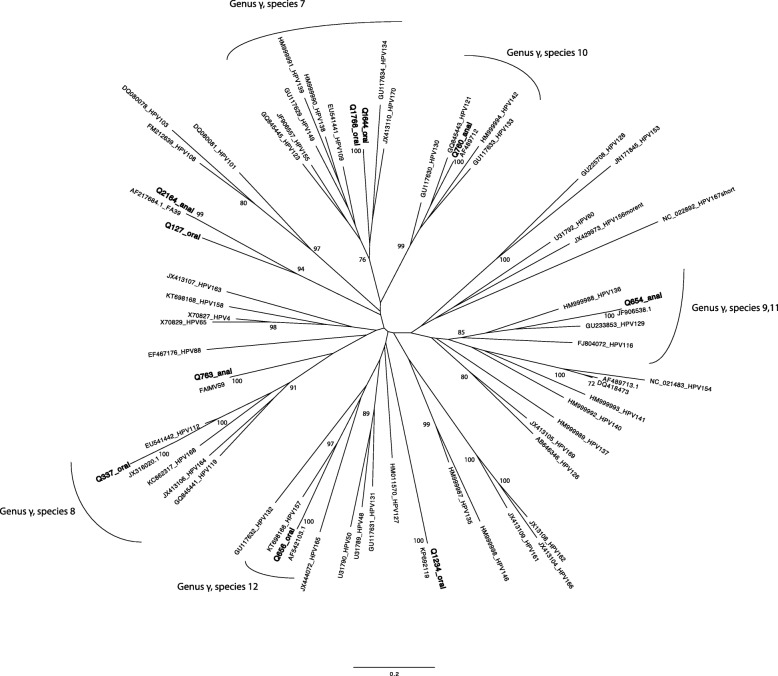
A maximum likelihood phylogenetic analysis was inferred on 10 unknown collected samples (bold) and 59 reference sequences using the GTRCAT model. Out of 10 unknown samples, 6 sequences were clustered within previously classified HPV species lineages. Bootstrap supports lower than 70% were excluded from the tree. GenBank accession number: MH647655- MH647663

For the phylogenetic analysis of our untyped HPV samples we used 10 partial cds sampled sequences from major capsid protein L1 FAP PCR products and 59 reference sequences. Our reference sequences represent 50 classified and 9 untyped HPV sequences (see Table 3). According to our phylogenetic analysis, 6 of our sampled sequences (Q1766, Q1644, Q760, Q337, Q656 and Q654) clustered with previously classified reference sequences from species 7, 10, 8, 12 and 9 –γ genus. Sequences Q763, Q1234, Q2164 and Q127 remained unclassified while showing great similarity with unclassified genomic regions AF489714 (FAMS9), KP692119, and AF217684.1 respectively (see Fig. 1 and Table 3).

**Table 3:** Untyped HPV strains detected by FAP59/64 primers

		Patient code	Closest strain	GenBank accession number	Nucleotide sequence similarity(%)
HIV positive	Anal swabs	Q654	Isolate SE17	JF906538.1	97%
		Q760	Isolate FA79	AF455142.1	99%
		Q763	Isolate FAIMVS9	AF489714.1	99%
		Q1539	Mixed^a^		
		Q2164 Q1354	Isolate FA39 MM7	AF217684.1 KC869667	97% 98%
	Oral Swabs	Q337	Isolate SE80	JX316020.1	91%
		Q656	Isolate FA97	AF542103.1	97%
		Q1017	Mixed^a^		
		Q1234	Isolate SE435	KP6922119.1	99%
		Q1644 Q1766	Isolate FA12.2 Isolate FA12.2	AY502596.1 AY502596.1	99% 99%
HIV negative		Q127	Isolate FA130	AY468427.1	99%

^a^Sanger sequence gave no clear results in this sample

## Discussion

In HNC, particularly oropharyngeal cancer, the true prevalence and involvement of HPV in the carcinogenetic process is still unknown. In fact, many studies report low prevalence of HPV obtained with systems used for the determination of HPV in cervical cancer screening (specific for α types), while other authors found higher prevalence when they used a platform able to detect HPV types belonging to genera other than α genus [4] both in oral cavity and in oral cancer [4, 39]. Agalliu recently found a correlation between the infection of some β, ɣ HPV types and OSCC [4]. Furthermore, a recent meta- analysis provided additional evidence of the involvement of β HPV types in the development of SCC in immunocompetent individuals [40]. Additionally, several mechanistic studies have consistently showed that E6 and E7 from several β HPV types are able to target cellular proteins, such as p53 and pRb, and to deregulate fundamental events involved in cellular transformation [41]. Thus β and ɣ HPV types detection could be included in a HPV screening test for oral cancer prevention, while a correct and suitable detection of HPV infection is becoming a priority. Currently, there are few studies that compare the HPV prevalence obtained using the protocols, which are normally for cervical cancer screening with those obtained using methods that are able to detect HPV types belonging to genera other than α or new ones. In this study, in order to assess the impact of PCR system in HPV prevalence determination, we carried out the detection of HPV with MY09/11 primer set, used in several genotyping molecular assays (for example Linear Array HPV genotyping Test, Roche Molecular system, CA USA; CLART HPV2 clinical arrays, Genomica, Madrid, Spain,) and FAP primers able to detect α, β, γ HPV types in several specimens. In oropharyngeal swabs samples, MY09/11 primer set was able to detect 39.4% (13/33) of HPV positive samples, while FAP set of primers detected the presence of HPV in 20/33 samples (60.6%). Beta HPV was the main genus detected with a prevalence of 69.7% in HPV positive samples. This prevalence was in agreement with Bottalico et al. that used FAP primer and MY09/11 for detecting HPV in oral swabs [14]. HPV5 was the most frequent β_1_ type and HPV145 the main type among β_2_ species as described by Bottalico [14]. HPV38, which was reported as the main β_2_ type in Forslund study [42], was absent in our samples. HR, namely HPV16, was observed only in one specimen from an HIV positive subject, whereas HR HPV were frequently observed in Bottalico study group [14]. The low frequency of HR could be imputed to immune status of the patients. Our patients were under HAART therapy and most of them had a CD4 T cell count > 200 /μl. No severe compromise of the immune-system could have limited a persistence of HR in this anatomical site. Unfortunately, no information was given in the Bottalico paper on patient CD4 T cell count, and this fact did not allow a correct comparison of these results. According to previous data reported by Forslund et al. [42], no difference in HPV prevalence was observed among men and women suggesting that host specific factors could contribute to HPV persistence and neoplasia development. Gamma genus represented 21.8% of HPV positive samples. To note, 6/7 γ strains were untyped. Phylogenetic analysis revealed that 2 samples (Q1766 and Q1644) belonged to γ_7_ species, while Q656 to γ 12 species and Q337 to γ_9_ species. Interestingly, the oral untyped genotypes fell in different genera and cluster with the anal untyped strains, suggesting a specific tropism of these gamma types for oral mucosa. Gamma 12 and 7 were the most representative γ species described by Bottalico in oral rinse and Agalliu in HNC, suggesting their potential involvement in OSCC development.

Unlike Forslund and Hampras [42, 43], we did not identified any β_3,_ β_4,_ β_5_ HPV types. This may be due to the types of biological samples that were used or to differences in the efficiency of extraction methods that were applied, which could have influenced the outcome of PCR [44]. Finally, differences in results might also be explained by the geographical distribution of HPV types. In general, differences in HPV β and γ types and their prevalence were observed by the Luminex platform. According to HIM study [43], among β_1_ genotypes, HPV12 and HPV5 were the prevalent genotypes. While, among β_2_ strains, HPV38 was the most represented,and it was never detected in our samples. Interestingly HR HPV38 was also the main β_2_ type observed also in Moscicki study, whereas HPV21 represented the main β_1_ type [45]. A different pattern of β types could be related to a different cellular input as described in a previous study [46], where several β HPV types showed essentially increasing prevalence with increasing DNA input. Beta HPV types 8, 14, 20, 21, 23, 38, and 92 showed increased prevalence only in higher DNA input groups [46]. This may impose compromises in comparisons between studies; for example, our HPV38 negative result could be explained by an insufficient DNA input in the PCR assay. Further research is needed to establish whether there is an influence between cell number input and HPV β detection in oropharyngeal anatomical site and which cut-off would be suggested to avoid false negative results. Possible type-specific differences in sensitivity to cellular input have to be evaluated in wider studies. If they are confirmed, they may be explained by the different sensitivities in detection systems and/or in the different viral load spectra. We detected 9 α strains in anal site FAP primers that had not been typed by Genomica, and 2 β_2_ types, HPV9 and HPV37, that had not been reported among oropharyngeal swabs. Βeta types frequency was sensibly lower to that found by Luminex system reported by Torres et al. [17] which found 65.6% of β types in anal swabs from HIV positive MSM and 59.1% among HIV negative MSM. In this study, HPV12 and HPV 107 were the prevalent types among β_1_ species, while HPV38 and HPV120 were the most frequently observed β_2_ types. Among γ genus, the γ_10_ species was the most prevalent in both MSM HIV positive and -negative groups, while we observed only γ untyped strains. Donà reported higher prevalence of β (27.6%) and γ types (29.3%) in a group of MSM similar to ours using the Luminex system [16]. Some hypothesis could put forth to explain these data: i) The Luminex system could be much more sensitive than the FAP system, or ii) that cross-reactivity could occur in β and γ types detection when α multiple infections are present. In literature, cross-reactivity was also observed among α genotypes. Preisler observed cross-reactivity both in LR and HR genotypes using HC2, COBAS, and APTIMA assays, despite what manufacturer claims: about 25% of HPVDNA results in primary screening accounting for cross-reactivity, regardless of the assay [47]. To obtain improved analytical and clinical performance, cross-reactivity studies should be focused, since this aspect could influence the effectiveness in a future head neck HPV cancer screening.

In cervical swabs the FAP primers mainly showed the presence of α genotypes (HPV90, α_14_; HPV32, α_1_) which were not detected by CLART HPV2 clinical array/MY09-11primers and 1 β_1_ type (HPV14). This low prevalence of β types confirms the data reported by Bottalico et al. [14], which found a prevalence of 1% of the β types and 3% of the γ types in the cervical samples, emphasizing a weak β and γ type tropism towards the female genital mucosa. However, the genotypes HPV93 and HPV124, detected by Bottalico in cervical specimens, were never observed among our cervical samples, suggesting a different geographic distribution of HPV β and γ type similarly to that described for α genus. Conversely, these findings were in disagreement with those obtained by Luminex system, as described in previous study [18].

A quantitative detection of the viral load in hair follicles demonstrated that the β genotype copy number was considerably lower than that reported for mucosal high-risk types from α genus in cervical tissue [48]. Thus, only comparable viral load detection studies with different methods and the potential for multiple infections detection could explain these differences in prevalence of β types, which are reported in literature.

Overall, our results confirmed a prevalence of > 20% for HPV strains in the oropharyngeal anatomical district. The genus β and γ were predominant when the analysis was carried out with FAP primers. The discrepancy on the prevalence and HPV types reported in other studies [16–18, 49] seems to be due to the detection system: highest prevalence was always obtained with the Luminex system. Different sensitivity and specificity of HPV detection methods were also a problem in the determination of α HPV types, as described in a systematic review where approximately 30–60% of all positive results showed discordance [50]. A global proficiency program like LabNet for α types in cervical HPV infection surveillance programs should be planned also for α, β and γ HPV type detection in oropharyngeal samples [51]. Standardizing methods for oral sample collection and HPV detection would ensure comparability between different detection methods in oral cavity samples [52, 53].

In addition, considering that γ genus has been growing rapidly (currently 98 γ types have been identified) surpassing α and β genera, and that 77% of the new types deposited in the HPV center within 2015 belonged to γ ​​genus [54–56], our data reinforces the relevance of using primer sets able to detect a wide spectrum of HPV strains including β and γ types as well as new genotypes for HPV detection in the oropharyngeal anatomical site. This seems to be a crucial point since the meta genomic approach applied in some analysis [13] should be used with caution, taking in account the possibility of having an overestimation of HPV types [57] and requiring a confirmation of positivity by the Sanger method.

## Additional file

Additional file 1 **Table S1** Study population characteristics of HIV infected patients. (DOCX 14 kb)
