# Supplementary material for: Alpha, Beta, gamma human PapillomaViruses (HPV) detection with a different sets of primers in oropharyngeal swabs, anal and cervical samples
Source: Virol J. 2019 Mar 4;16:27. doi: 10.1186/s12985-019-1132-x (PMC6398256; doi:10.1186/s12985-019-1132-x)
Supplement: Supplementary file 1 — Table S1 Study population characteristics of HIV infected patients. (DOCX 14 kb) [file 12985_2019_1132_MOESM1_ESM.docx]

**Table S1_supplementary data.**

Study population characteristics of HIV infected patients.

|  | | |  |
| --- | --- | --- | --- |
| **Oral Swabs** | HIV + | Total patients | 55 |
|  |  | CD4 T cell* | 532.3 ±275.6 |
|  |  | Viral load^ | 169.0±130.0 |
|  |  | Age° | 46  (range 26-65) |
|  |  |  |  |
| **Anal Swabs** | HIV+ | **Total patients** | 186 |
|  |  | **CD4 T cell** | 690.7 ±343.9 |
|  |  | **HIV RNA** | Not detected(n=104) |
|  |  |  |  |
|  |  |  | Detected (n=82)  320.7 ±809.4 |
|  |  | **Age** | 42  (range 23-68) |
| **Cervical Swabs** | HIV+ | **Total Patients** | 43 |
|  |  | **CD4 T cell** | 539.8 ±230.3 |
|  |  | **HIV RNA** | Not detected (n=23) |
|  |  |  | Detected (n=20)  550.0 ±100.6 |
|  |  | **Age** | 41  (range 24-64) |

*Mean (±standard deviation) expressed as cells/µL; ^ mean (±standard deviation) expressed as copies/mL; °median value (range); n, number.
